# From incidental to intentional: ultrasound imaging in detecting internal jugular vein duplication/fenestration from a case report

**DOI:** 10.1097/MD.0000000000042935

**Published:** 2025-06-27

**Authors:** Junda Cao, Na Lei, Qin Gong, Huihui Bao, Honghua Zhou, Yanping Zhao

**Affiliations:** a Jiujiang City Key Laboratory of Cell Therapy, Department of Cardiovascular, The First Hospital of Jiujiang City, Jiujiang, China; b Department of Cardiovascular Medicine, The Second Affiliated Hospital, Jiangxi Medical College, Nanchang University, Nanchang, China; c Department of Disinfection and Supply, General Hospital of Central Theater Command of the People’s Liberation Army, Wuhan, China; d Department of Vascular Surgery, The First Affiliated Hospital of Nanchang University, Nanchang, China; e Department of General Surgery, Jiujiang University Affiliated Hospital, Jiujiang, China.

**Keywords:** ultrasound, vascular malformation, vein angiography, vein duplication, vein fenestration

## Abstract

**Rationale:**

Few reports in the literature describe internal jugular vein fenestration or duplication, and these are typically incidental surgical findings, which include a few 3-dimensional radiological images of these anomalies obtained from preoperative imaging. The potential of ultrasound has been greatly underestimated because there is no previous literature on ultrasound-based diagnosis of this disease.

**Patient concerns:**

A 40-year-old woman presented with dizziness. A computed tomography arteriography examination of the head and neck at another hospital found no abnormalities. The magnetic resonance imaging of the head also showed no abnormalities, and the echocardiogram was normal. Psychological tests revealed no significant anxiety.

**Diagnoses:**

Vascular ultrasound revealed duplication/fenestration of the internal jugular vein, and jugular venography ultimately confirmed this vascular malformation. As a result, she was diagnosed with internal jugular vein duplication/fenestration.

**Interventions:**

Considering the current symptoms did not affect her quality of life, and this vein malformation did not significantly increase the risk of other diseases, she was placed on a clinical observation regimen.

**Outcomes:**

Her symptoms did not worsen. After 2 years of follow-up, there had been no change in her jugular vascular malformations.

**Lessons:**

Understanding internal jugular vein duplication and fenestration is crucial for clinicians, particularly those involved in head and neck therapy, as these anomalies can significantly affect surgical outcomes and patient safety. Preoperative imaging techniques, such as ultrasound, can aid in identifying these variations, allowing for improved surgical planning and risk management.

## 1. Introduction

Fenestrations and duplications of the internal jugular vein (IJV) are rare anatomical variations that can have significant implications during surgical procedures and central venous access.^[1]^ It can lead to complications if not properly identified preoperatively. Spinal accessory nerve passing, and other malformations added the complexity of such anatomical variations to surgical interventions.^[1]^ In addition, this malformation has variations in morphology that can complicate catheterization and increase the risk of accidental injury during surgical procedures. Preoperative imaging techniques, such as ultrasound, can aid in understanding this variation, which is crucial for clinicians, especially those involved in head and neck therapy.

## 2. Case report

**Figure 1.**
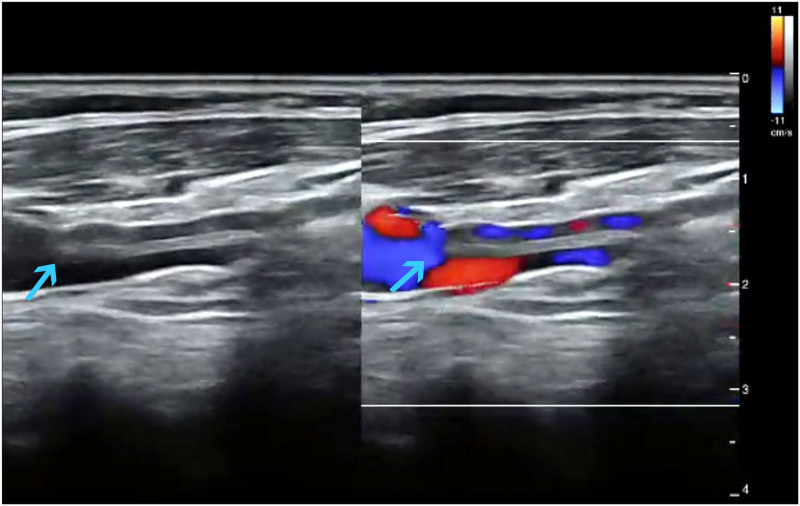
Ultrasound images of blood vessels in the neck revealed the internal jugular vein fenestration/duplication on a 5 to 8 MHz high-frequency probe (the arrows indicate the types of venous duplication).

**Figure 2.**
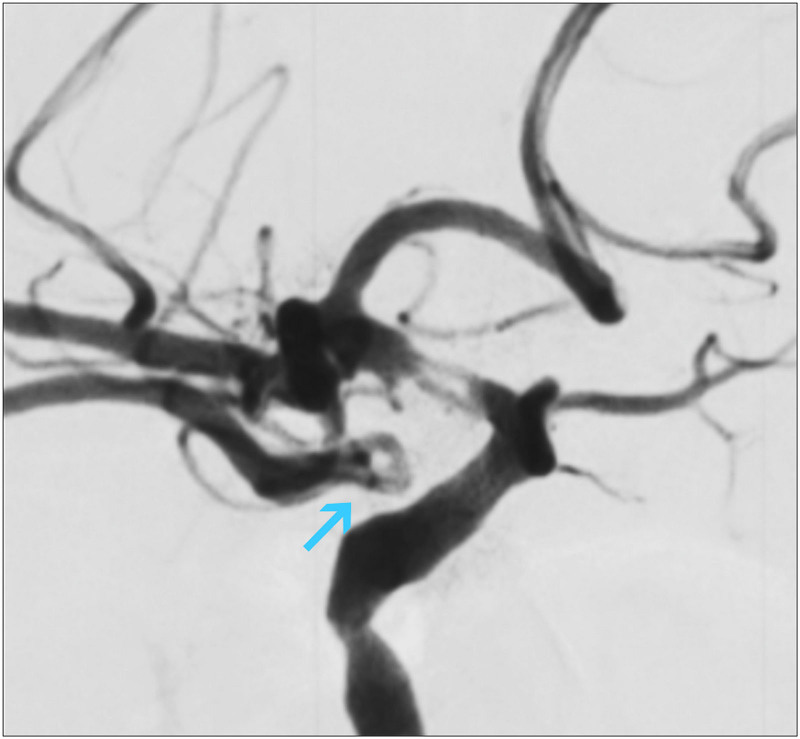
Internal jugular vein fenestration/duplication confirmed by internal jugular vein angiography, demonstrating 2 lumens divided by a septum (arrows).

A 40-year-old woman was hospitalized for intermittent dizziness. The duration of this symptom is >10 years and has no significant effect on the menstrual cycle. The patient had no previous hypertension, diabetes, or heart disease. Magnetic resonance examination of the head showed no significant abnormalities. On physical examination, her body temperature was 36.6°C, her pulse rate was 72 beats/min, and her respiration rate was 13 breaths/min. Her blood pressure was 113/72 mm Hg. Her breathing sounds, heart sounds, and bowel sounds are normal. The physical examination of the thyroid was normal, and palpation or auscultation of the neck vessels also revealed no abnormal findings. No anatomical abnormalities or pathological changes were found in the cephalic arterial angiography. And on a vascular ultrasound of her neck, abnormal anatomical imaging was found, where a spectated echogenic structure in the right distal jugular lumen (Fig. 1). The septate divides the venous lumen into 2 lumens in which the same direction flow signal can be observed, and the 2 ends are not closed (Fig. 1). Finally, the deformity was confirmed by invasive angiography (Fig. 2). Considering the current symptoms did not affect her quality of life, and this vein malformation did not significantly increase the risk of other diseases, she was placed on a clinical observation regimen. Her symptoms did not worsen, and vascular ultrasound showed no change in the anomaly during the 2-year follow-up.

## 3. Discussion

The IJV is a crucial vessel in the head and neck region, and its anatomical variations, including fenestration and duplication, can have significant implications for surgical procedures and diagnostic interventions. Internal jugular venous duplication is a bifurcation of the veins into 2 branches, with each branch separately connected to the subclavian vein.^[2,3]^ The fenestration deformity of the IJV is a bifurcation of the middle part of the vein that converges into a branch before joining the subclavian vein.^[2,3]^ However, the 2 are often used interchangeably in the literature and are not strictly distinguished.^[4,5]^

Understanding the IJV abnormalities is important to avoid iatrogenic injury during neck surgery (e.g., neck cancers such as oral cancer and thyroid cancer are often treated with prophylactic and therapeutic neck sampling or dissection) and central vein catheterization. A study reported the potential risk of iatrogenic injury to the vasculature and the spinal accessory nerve during surgical interventions.^[6–9]^ The presence of IJV duplication or fenestration can complicate surgical procedures by increasing the risk of inadvertent vascular injury. For example, during neck dissection, failure to recognize these anomalies can lead to unexpected hemorrhage, particularly if the superficial branch of a duplicated vein is damaged.^[6]^

The etiology of IJV duplication/fenestration deformity has garnered attention in recent anatomical and clinical studies. The underlying causes of these anatomical variations remain unclear, but they may be attributed to developmental anomalies during embryogenesis. Studies have suggested that variations in venous drainage patterns and the presence of external compressive forces could contribute to the formation of these deformities.^[4]^ In addition, the presence of fenestrations in the IJV has been associated with other vascular anomalies, indicating a potential link between these conditions.^[4]^

Recent studies have highlighted the rarity of IJV fenestration/duplication, with reported incidences ranging from 0.4% to 3.3%.^[6]^ If anatomical details must be emphasized, these IJV malformations include duplication, bifurcation, fenestration, posterior tributary, and trifurcation.^[10]^ In over two-thirds of the patients, these IJV malformations were observed unilateral and on the left side.^[10]^ Evidence indicates that the right IJV is somewhat larger and thicker than the left.^[11]^ Fortunately, it is widely known that the right IJV is a favored site for venous access because of the carotid artery’s accessibility and less intimate nature.

The vast majority of IJV fenestration/duplication patients are asymptomatic, but a small number of patients present with neck swelling and difficulty breathing and swallowing, the potential for complications necessitates careful evaluation and management of patients with such vascular anomalies.^[12]^ Another study reported misdiagnosis of IJV malformations as branchial cleft cysts.^[13,14]^ The absence of symptoms does not mean there is no harm. The anatomical variations of the IJV, including fenestration and duplication, can have implications for venous drainage and may contribute to the development of conditions like chronic cerebrospinal venous insufficiency.^[15–17]^

These vascular malformations were discovered incidentally during neck dissection in the existing literature. Because current computed tomography angiography primarily focuses on arteries, discussing the optimal diagnostic tool for venous anomalies like IJV duplication is necessary. Of course, there is no doubt that angiography is the gold standard for diagnosing vascular malformations. However, ultrasound has emerged as a valuable diagnostic tool for identifying IJV abnormalities with noninvasive or radiation-free. For instance, Doppler ultrasonography can effectively assess the morphology and hemodynamics of the IJV, aiding in the detection of conditions such as IJV valve incompetence and thrombosis. In addition, the significance of this finding, which had not been previously documented, the use of color Doppler ultrasonography has been instrumental in revealing IJV duplication in patients with multiple sclerosis.

## 4. Conclusion

In conclusion, understanding IJV duplication and fenestration is crucial for clinicians, particularly those involved in head and neck therapy, as these anomalies can significantly affect surgical outcomes and patient safety. Preoperative imaging techniques, such as ultrasound, can aid in identifying these variations, allowing for improved surgical planning and risk management.

## Acknowledgments

The authors thank the patients who agreed to be included in this study.

## Author contributions

**Data curation:** Junda Cao, Qin Gong, Honghua Zhou, Yanping Zhao.

**Methodology:** Junda Cao, Na Lei, Honghua Zhou.

**Writing – original draft:** Junda Cao, Qin Gong, Honghua Zhou.

**Funding acquisition:** Na Lei, Yanping Zhao.

**Investigation:** Na Lei, Qin Gong.

**Software:** Na Lei, Honghua Zhou, Yanping Zhao.

**Writing – review & editing:** Na Lei, Yanping Zhao.

**Resources:** Qin Gong, Huihui Bao, Honghua Zhou.

**Supervision:** Qin Gong, Huihui Bao, Yanping Zhao.

**Conceptualization:** Yanping Zhao.

**Project administration:** Yanping Zhao.

**Validation:** Yanping Zhao.

## References

[1] Guarino P Tesauro P Giordano L Caporale CD Presutti L Mattioli F. Surgical and radiological perspectives for the spinal accessory nerve passing through a fenestrated internal jugular vein: case series and literature review. J Surg Case Rep. 2024;2024:rjae099. 38617811 10.1093/jscr/rjae099 PMC11014881

[2] Burman S Pandey S Rao S Rao S. Duplication of the internal jugular vein: a rare presentation during neck dissection. BMJ Case Rep. 2021;14:e239007. 10.1136/bcr-2020-239007 PMC7896602 33602762

[3] Aladham Y Momin SMB Ahmed O Jackson S. Internal jugular vein fenestration: an intraoperative finding without a radiological clue. Cureus. 2022;14:e21166. 35165616 10.7759/cureus.21166 PMC8831423

[4] Rusu MC Vrapciu AD Popescu SA. High bilateral fenestration of the internal jugular vein. Surg Radiol Anat. 2022;44:703–8. 35396939 10.1007/s00276-022-02931-w

[5] Wang X Peng L Guo H. Internal jugular vein fenestration and duplication: anatomical findings, prevalence, and literature review. Front Surg. 2020;7:593367. 33282909 10.3389/fsurg.2020.593367 PMC7691239

[6] Bachoo I Evans B. Duplication of the lower third of the internal jugular vein - case report and surgical implications. Br J Oral Maxillofac Surg. 2014;52:772–3. 25074338 10.1016/j.bjoms.2014.05.016

[7] Zhang G Chen R Ghorbani H. Artificial intelligence-enabled innovations in cochlear implant technology: advancing auditory prosthetics for hearing restoration. Bioeng Transl Med. 2025;10:e10752. 40385537 10.1002/btm2.10752 PMC12079510

[8] Ding H Wang C Ghorbani H. The impact of magnesium on shivering incidence in cardiac surgery patients: a systematic review. Heliyon. 2024;10:e32127. 38873687 10.1016/j.heliyon.2024.e32127 PMC11170178

[9] Ghorbani H Minasyan A Ansari D. Anti-diabetic therapies on dental implant success in diabetes mellitus: a comprehensive review. Front Pharmacol. 2024;15:1506437. 39723258 10.3389/fphar.2024.1506437 PMC11668599

[10] Mumtaz S Singh M. Surgical review of the anatomical variations of the internal jugular vein: an update for head and neck surgeons. Ann R Coll Surg Engl. 2019;101:2–6. 30322289 10.1308/rcsann.2018.0185 PMC6303832

[11] Saiki K Tsurumoto T Okamoto K Wakebe T. Relation between bilateral differences in internal jugular vein caliber and flow patterns of dural venous sinuses. Anat Sci Int. 2013;88:141–50. 23572397 10.1007/s12565-013-0176-z PMC3654179

[12] Kirmani S Rashid M Ali I Badar F. External jugular vein aneurysm: a rare cause of neck swelling. J Ultrasound Med. 2011;30:1157–8. 21795494 10.7863/jum.2011.30.8.1157

[13] Madoure A Penubarthi LK Kushwaha A Alexander A. Unilateral fenestration of internal jugular vein with a radiological clue: a rare case report and literature review. Cureus. 2023;15:e39863. 37404430 10.7759/cureus.39863 PMC10315060

[14] Nayak SB. Jug handle tributary of internal jugular vein: a potentially dangerous venous variation. J Craniofac Surg. 2019;30:e7–8. 30277954 10.1097/SCS.0000000000004826

[15] Torres C Hogan M Patro S. Extracranial venous abnormalities: a true pathological finding in patients with multiple sclerosis or an anatomical variant? Eur Radiol. 2017;27:239–46. 27011374 10.1007/s00330-016-4314-6

[16] Nayak SB. Split internal jugular vein: surgical and radiological implications. Br J Oral Maxillofac Surg. 2017;55:870–1. 10.1016/j.bjoms.2017.08.002 28843969

[17] Nayak BS. Surgically important variations of the jugular veins. Clin Anat. 2006;19:544–6. 16372344 10.1002/ca.20268

